# Intranasal Delivery of Proenkephalin-Expressing Differentiated Mesenchymal Stem Cells Attenuates Morphine Dependence Behaviors and Modulates Neurotrophic Factors in Rats

**DOI:** 10.1080/17590914.2026.2739826

**Published:** 2026-10-06

**Authors:** Mohammad Saleh Ranaiy, Hamed Ghazvini, Seyedeh Masoumeh Seyedhosseini Tamijani, Mozhgan Abasi, Seyed Abbas Mousavi, Hamid Kalalian-moghaddam, Raheleh Rafaiee

**Affiliations:** a Student Research Committee, School of Advanced Technologies in Medicine, Mazandaran University of Medical Sciences, Sari, Iran; b Department of Neuroscience, School of Advanced Technologies in Medicine, Mazandaran University of Medical Sciences, Sari, Iran; c Department of Applied Cellular Sciences, School of Advanced Technologies in Medicine, Mazandaran University of Medical Sciences, Sari, Iran; d Department of Psychiatry, School of Medicine, Mazandaran University of Medical Sciences, Sari, Iran; e Center for Health Related Social and Behavioral Sciences Research, Shahroud University of Medical Sciences, Shahroud, Iran; f Psychiatry and Behavioral Sciences Research Center, Addiction Institute, Mazandaran University of Medical Sciences, Sari, Mazandaran, Iran

**Keywords:** Cell- and tissue-based therapy, morphine, proenkephalin, stem cells

## Abstract

Substance use disorder is a chronic relapsing condition characterized by persistent drug seeking, cognitive impairment, and neurobiological alterations in brain reward and memory circuits. Emerging evidence suggests that dysfunction of the endogenous opioid system and reduced neurotrophic signaling contribute to the pathophysiology of opioid dependence. Cell based therapeutic strategies may provide a novel approach to modulate these neural pathways. In the present experimental study, morphine dependence was induced in adult male rats using an escalating dose regimen. Animals were randomly allocated into control, morphine-dependent vehicle, and cell-treated groups. Proenkephalin-expressing differentiated mesenchymal stem cells (PENK-MSCs) were delivered via the intranasal route. Behavioral assessments were performed using conditioned place preference, novel object recognition, and the elevated plus maze to evaluate addiction related behaviors, cognitive performance, and anxiety like responses. Hippocampal brain derived neurotrophic factor (BDNF) expression was examined using immunohistochemistry. Morphine dependent rats exhibited significant behavioral alterations, including enhanced drug related preference, impaired recognition memory, and increased anxiety like behavior compared with control animals. In addition, hippocampal BDNF expression was significantly reduced following morphine exposure. Intranasal cell delivery significantly attenuated morphine-induced preference, reversed recognition memory deficits, mitigated anxiety-like behaviors, and upregulated hippocampal BDNF expression compared with the vehicle-treated morphine group. These findings demonstrate that intranasal delivery of PENK-MSCs attenuates behavioral deficits and promotes neurotrophic recovery in morphine-dependent rats. This approach may represent a promising experimental strategy for targeting neural mechanisms involved in substance use disorder, although further studies are required to confirm its long-term efficacy and translational potential.

## Graphical Abstract

**Figure:**
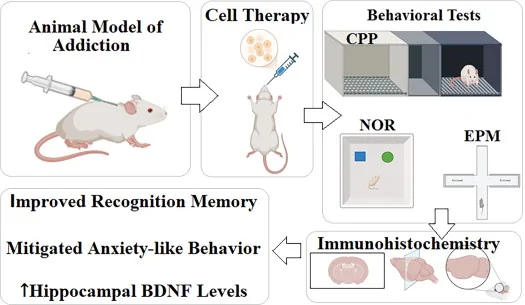

## Introduction

Drug use represents a major global public health challenge. In 2021, approximately 5.8% of the world population aged 15–64 years reported using illicit drugs, with nearly 60 million individuals consuming opioids (Volkow & Blanco, 2023). Achieving sustained abstinence remains difficult, as relapse is a common outcome during the withdrawal period (Melemis, 2023). Chronic drug exposure is also associated with a range of adverse effects, including neurocognitive impairments and other long-term complications that may further increase the risk of relapse (G. F. Koob et al., 2023). In individuals with substance use disorders (SUDs), repeated drug exposure induces persistent craving and compulsive drug-seeking behavior through alterations in the brain reward circuitry (Mohseni et al., 2022). In addition to psychological dependence, prolonged drug use can lead to physical dependence, characterized by the emergence of withdrawal symptoms upon cessation of drug intake (Badshah et al., 2024).

In addition to exogenous opioids, the brain contains an endogenous opioid system that plays a critical role in regulating reward, motivation, and pain processing. Endogenous opioid peptides are mainly classified into three families: endorphins, dynorphins, and enkephalins. These peptides act as neuromodulators by binding to opioid receptors and modulating neuronal signaling in multiple brain regions (Ghelardini et al., 2015). Among them, enkephalins are pentapeptides derived from proenkephalin and exist in two forms, met-enkephalin and leu-enkephalin. Enkephalins are widely distributed in the nervous system and influence several neurotransmitter systems involved in reward processing, including dopamine, GABA, glutamate, and acetylcholine (G. F. Koob & Volkow, 2016). Evidence suggests a close interaction between the enkephalinergic and dopaminergic systems, particularly within brain reward circuits (L. G. Rysztak & E. M. Jutkiewicz, 2022). Activation of opioid receptors, especially μ and δ subtypes, can modulate dopamine release in key regions such as the nucleus accumbens and ventral tegmental area, which are central components of the neural circuitry underlying SUDs (Listos et al., 2019).

Given the central role of neurobiological alterations in reward circuitry in SUDs, the development of novel therapeutic strategies targeting neural repair has gained increasing attention. In this context, stem cell-based therapies have emerged as a promising approach for neurological disorders characterized by circuit dysfunction and neuroplastic deficits. Delivered stem cells promote neural repair through multifaceted mechanisms, including lineage-directed differentiation and the paracrine secretion of neurotrophic factors that support synaptic plasticity and neuronal survival (Xiong et al., 2021). Although cell therapy has been investigated in several neurological conditions, only a limited number of studies have explored its potential in SUDs. For example, transplantation of bone marrow–derived mesenchymal stem cells (BM-MSCs) has been shown to alleviate alcohol-associated cognitive impairment in rodents through the reduction of oxidative stress and the release of trophic factors (Yang et al., 2015). Similarly, intravenous delivery of neural stem cells improved cognitive deficits and increased synaptic protein density in brain regions such as the hippocampus, amygdala, and anterior cingulate cortex in a model of fetal alcohol spectrum disorder (Shirasaka et al., 2012). In addition, administration of BM-MSCs has been reported to attenuate methamphetamine-induced brain damage and reduce relapse-related behaviors in rodents (Rafaiee et al., 2023). These studies have primarily focused on the side effects of alcohol (Camarini, 2019; Shirasaka et al., 2012) and methamphetamine use (Rafaiee et al., 2023). Notably, most previous studies have used undifferentiated stem cells, which may carry risks such as tumorigenesis and unpredictable differentiation within the host brain. In contrast, transplantation of differentiated neural cells may offer a more targeted therapeutic strategy by restoring specific neuronal populations and modulating defined neural circuits involved in addiction (Ranaiy et al., 2024).

Chronic exposure to exogenous opioids has been shown to suppress endogenous enkephalin production within reward-related brain pathways through negative feedback mechanisms (Ali et al., 2021). Given the advantages of targeted cell-based interventions, restoring endogenous enkephalinergic tone via the non-invasive intranasal route represents an innovative strategy to modulate opioid-compromised neural circuits. However, whether intranasally delivered proenkephalin-expressing differentiated mesenchymal stem cells (PENK-MSCs) can effectively mitigate morphine-induced behavioral adaptations remains to be elucidated. Therefore, the present study aimed to evaluate whether intranasal delivery of PENK-MSCs could attenuate reward-related behaviors, recognition memory deficits, and anxiety-like responses in a rat model of morphine dependence. Furthermore, we investigated whether this cell-based approach modulates hippocampal brain-derived neurotrophic factor (BDNF) expression as an underlying marker of neuroplastic recovery.

## Materials and Methods

### Animals

This study was conducted using 24 adults male Wistar rats weighing 180–220 g. The animals were obtained from the Animal House of Royan Institute and housed under controlled environmental conditions (temperature 23 ± 2 °C, humidity 50%–60%, and a 12 h light/dark cycle). Rats had free access to standard laboratory chow and water ad libitum. All animals were allowed to acclimatize to the housing conditions for one week prior to the experiments. Experimental procedures were performed during the light phase between 08:00 and 15:00. All procedures were conducted in accordance with the National Institutes of Health Guide for the Care and Use of Laboratory Animals and were approved by the Institutional Animal Ethics Committee of Mazandaran University of Medical Sciences.

## Drugs

The following drugs were used in this study: morphine sulfate (Darou Pakhsh, Tehran, Iran), ketamine and xylazine (Alfasan, Woerden, Netherlands), and naloxone hydrochloride (Caspian Tamin Pharmaceutical Co., Rasht, Iran).

## Experimental Protocol

This study included three experimental groups (*n* = 8 per group) as follows

- Control group (CON): Rats in this group received no morphine administration or intranasal treatment and were maintained under identical housing and handling conditions throughout the experimental period.
- Morphine + intranasal PBS group (Sham): Rats received subcutaneous morphine for three weeks followed by intranasal administration of phosphate-buffered saline (PBS) as a vehicle treatment.
- Morphine + intranasal cell-treated group (PENK): Rats received subcutaneous morphine for three weeks followed by intranasal delivery of PENK-MSCs.

Morphine dependence was induced using morphine sulfate solution. The drug was administered subcutaneously twice daily at 08:00 and 20:00 according to a gradually escalating dosing schedule. Animals received 5 mg/kg from days 1 to 5, 10 mg/kg from days 6 to 10, 15 mg/kg from days 11 to 15, and 20 mg/kg from days 16 to 21 (Gheibi et al., 2025). Three days after the final morphine administration, intranasal delivery of either vehicle (PBS) or PENK-MSCs was performed as described in the subsequent sections. All animals subsequently underwent behavioral testing. The overall experimental timeline, behavioral assessments, and molecular analyses are illustrated in Figure 1.

**Figure 1.**
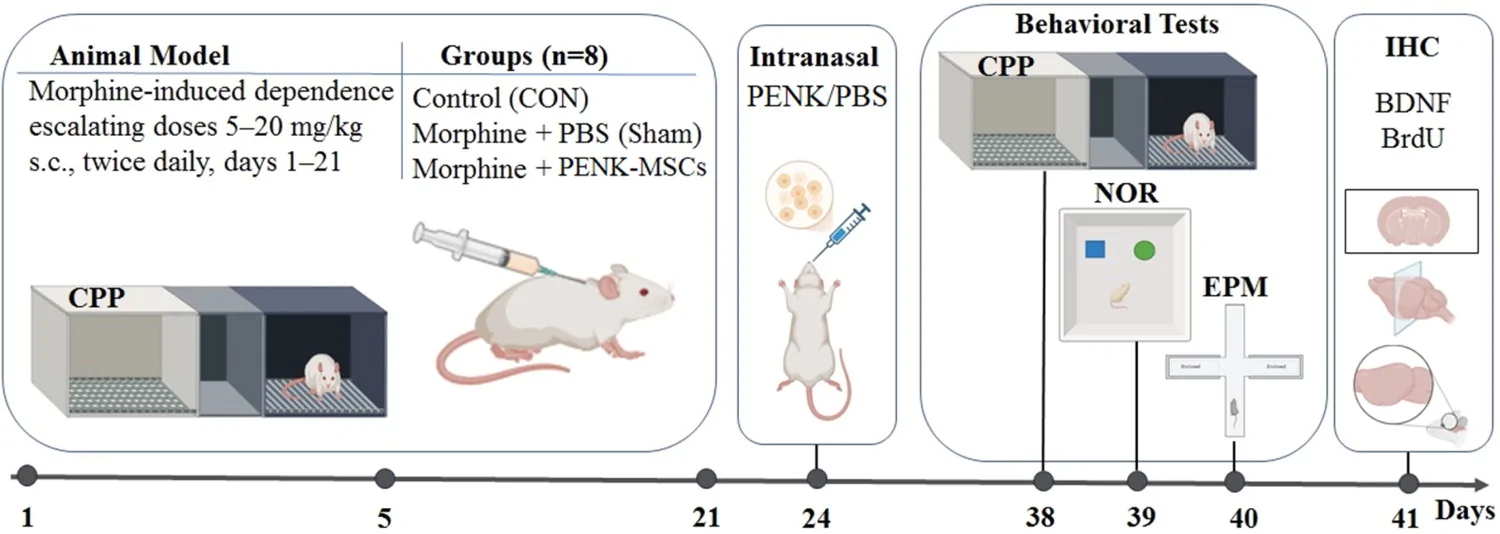
Timeline of experimental procedures, behavioral assessments, and histological measures. Abbreviations: BDNF, brain derived neurotrophic factor; CPP, conditioned place preference; PENK, proenkephalin-expressing differentiated stem cells; EPM, elevated plus maze; IHC, immunohistochemistry; NOR, novel object recognition; PBS, phosphate buffered saline; s.c., subcutaneous (created by BioRender.com).

### Neuronal Differentiation of Mesenchymal Stem Cells and Proenkephalin Characterization

The BM-MSCs used in this study were obtained from the laboratory’s existing cryopreserved cell repository. These MSCs had been previously isolated from the femurs and tibias of two-month-old male Wistar rats (approximately 200 g) following established protocols. To maintain experimental consistency, a characterized cell bank at passage 3 had been cryopreserved in liquid nitrogen. The MSCs identity was verified via flow cytometry, confirming the positive expression of CD73, CD90, CD105, and CD166, and the absence of CD34 and CD45, consistent with previously documented characterization (Rafaiee et al., 2023). For the present experiments, these cells were thawed and cultured in DMEM supplemented with 10% fetal bovine serum and 1% penicillin-streptomycin at 37 °C in a humidified atmosphere containing 5% CO_2_. Cells were expanded to passage 4 prior to the initiation of the neuronal differentiation protocol.

To induce neuronal differentiation, MSCs at 70%–80% confluence were transitioned into a specific neuronal differentiation medium. The basal medium consisted of DMEM/F12 (1:1) supplemented with 10% fetal bovine serum (FBS), 1% insulin-transferrin-selenium (ITS) supplement, 10 ng/mL basic fibroblast growth factor (bFGF), and 0.5 µM all-trans retinoic acid (RA). The differentiation medium was refreshed every three days to maintain nutrient stability and growth factor activity. This induction process was carried out for a total of 12 days at 37 °C in a humidified 5% CO_2_ atmosphere. Neuronal lineage commitment and differentiation into a proenkephalinergic phenotype were confirmed by quantifying the mRNA expression levels of proenkephalin (PENK) and the neural transcription factor Ikaros using quantitative real-time PCR (qRT-PCR), as previously described (Hafizi et al., 2012).

## Labeling and Preparing Cells for Administration

To enable identification of transplanted cells in brain tissue, differentiated cells were labeled with 10 µM 5-bromo-2-deoxyuridine (BrdU). BrdU was added to the culture medium 72 hours prior to transplantation. Cell viability was assessed using the trypan blue exclusion method and viable cells were counted using a hemocytometer (Rafaiee et al., 2023).

The cells were then trypsinized, centrifuged, and resuspended in culture medium at the required concentration. Approximately 2.5 × 10^5^ viable differentiated cells were prepared for in vivo delivery. The administration procedure was performed under general anesthesia, and the cell suspension was delivered intranasally as established previously (Rafaiee et al., 2023). Following cell administration, the animals were monitored and behavioral assessments were conducted two weeks later (Rafaiee et al., 2023). At the end of the experiment, histological analyses were performed to evaluate the survival and localization of transplanted cells and BDNF in the left hippocampus.

## Intranasal Administration Protocol

Intranasal administration was performed under general anesthesia using a stereotaxic apparatus to maintain a stable head and neck position. Anesthesia was induced by intraperitoneal injection of ketamine (100 mg/kg) and xylazine (10 mg/kg). The animal’s head was then positioned at an angle of approximately 70–90 degrees to facilitate optimal delivery through the nasal cavity (Rafaiee et al., 2023). A pediatric plastic angiocatheter needle was gently placed at the entrance of each nostril, and the cell suspension was administered dropwise using a calibrated micropipette (1–10 µL). Each application consisted of approximately 3–6 µL to ensure adequate absorption through the nasal cavity while minimizing the risk of aspiration. Volumes lower than this range are insufficient for effective delivery to the olfactory epithelium, whereas larger volumes may enter the nasopharynx and potentially cause respiratory distress during anesthesia. The total administered volume was 24 µL, delivered gradually to allow proper intranasal absorption. After administration, the animals were maintained in the same head elevated position under anesthesia for approximately one hour to facilitate nasal absorption. Once fully recovered from anesthesia, the animals were returned to their home cages. In the sham group, PBS was administered intranasally using the same procedure and volume.

## Behavioral Assessments

Behavioral testing was performed two weeks after cell transplantation. All animals underwent the same sequence of behavioral tests, including conditioned place preference (CPP), novel object recognition (NOR), and elevated plus maze (EPM), to evaluate reward-related behavior, recognition memory, and anxiety-like behavior, respectively. All behavioral tests were conducted during the light phase between 09:00 and 14:00.

## Conditioned Place Preference

The CPP was tested in a three-part Plexiglas box (30 × 40 × 30 cm^3^). The procedure consisted of a pre-test session, a three-day conditioning phase, and a final test session.

### Pre-Test (Familiarization)

Before induction of the addiction model, each rat was placed in the neutral compartment and allowed to freely explore all three compartments for 15 minutes. The time spent in each compartment was recorded, and the initially preferred and non-preferred compartments were determined for each animal.

### Conditioning Phase

During the conditioning phase (days 2 to 4), rats received morphine injections and were confined to the previously non-preferred compartment for 45 minutes. Six hours later, the animals received distilled water as the vehicle and were confined to the preferred compartment for 45 minutes. This conditioning procedure was repeated for three consecutive days.

### Test Session

On day 5, animals were tested for CPP under drug-free conditions. Each rat was placed in the apparatus and allowed to freely explore all compartments for 10 minutes. The time spent in each compartment was recorded. The CPP score was calculated as the difference between the time spent in the drug-paired compartment and the time spent in the non-drug-paired compartment (Rafaiee et al., 2023).

## Novel Object Recognition Test

Recognition memory was evaluated using the NOR test in a cube-shaped arena (40 × 50 × 50 cm). The test consisted of three phases: habituation, training, and testing.

### Habituation Phase

Each rat was placed in the empty arena for 10 minutes to freely explore the environment. This procedure was repeated for two consecutive days to allow habituation to the testing environment.

### Training Phase

During the training session, two identical objects (A and A) were placed in two adjacent corners of the arena. Each rat was allowed to explore the objects for 10 minutes. Exploration was defined as directing the nose toward the object at a distance of less than 2 cm or touching the object with the nose. Sitting on or leaning against the object was not considered exploratory behavior. The time spent exploring each object was recorded.

### Testing Phase

In the testing session, one of the familiar objects was replaced with a novel object (A and B). The rat was allowed to explore the objects for 10 minutes. The positions of the objects were alternated between animals to eliminate spatial bias. After each trial, the arena and objects were cleaned with 70% ethanol to remove olfactory cues. The discrimination index was calculated as: (time spent exploring the novel object minus time spent exploring the familiar object) divided by the total exploration time.

## Elevated Plus Maze

Anxiety-like behavior was assessed using the EPM. The apparatus consisted of two open arms (10 × 50 cm) and two closed arms (10 × 50 × 40 cm) extending from a central platform (10 × 10 cm). The maze was elevated 50 cm above the floor. Each rat was placed on the central platform facing an open arm and allowed to explore the maze for 5 minutes. The time spent in the open arms and the number of entries into the open arms were recorded as indices of anxiety-like behavior.

## Real-Time Quantitative PCR

The expression levels of proenkephalin (PENK) and Ikaros genes in differentiated enkephalinergic cells were evaluated using real-time quantitative polymerase chain reaction (qRT-PCR). Total RNA was isolated from cultured cells using RNX-Plus reagent (CinnaGen, Iran) according to the manufacturer’s protocol based on the chloroform–isopropanol extraction method. The concentration and purity of the extracted RNA were determined spectrophotometrically (NanoDrop, Thermo Fisher Scientific, USA) by measuring absorbance at 260 and 280 nm. Only RNA samples with an A260/A280 ratio between 1.8 and 2.0 were used for further analysis.

Complementary DNA (cDNA) was synthesized from 1 µg of total RNA using a reverse transcription kit (Thermo Fisher Scientific, USA) following the manufacturer’s instructions. Real-time PCR amplification was performed using SYBR Green PCR Master Mix (Applied Biosystems, USA) on a StepOnePlus Real-Time PCR System (Applied Biosystems, USA).

Each PCR reaction was carried out in a final volume of 20 µL containing 10 µL of SYBR Green master mix, 1 µL of forward primer, 1 µL of reverse primer, 2 µL of cDNA template, and 6 µL of nuclease-free water. The amplification protocol consisted of an initial denaturation step at 95 °C for 10 minutes, followed by 40 cycles of denaturation at 95 °C for 15 seconds, annealing at 60 °C for 30 seconds, and extension at 72 °C for 30 seconds. A melting curve analysis was performed at the end of each run to confirm the specificity of the amplified products.

Glyceraldehyde-3-phosphate dehydrogenase (GAPDH) was used as the internal reference gene to normalize gene expression levels. All reactions were performed in triplicate. Relative gene expression levels were calculated using the comparative Ct method (2^-ΔΔCt). The obtained values were used for subsequent statistical analysis. The primer sequences used for quantitative real-time PCR were adopted from (Ref. 18) (Hafizi et al., 2012) and presented in Table 1.

**Table 1.** Primer sequences used for quantitative real-time PCR analysis.

Gene	Forward primer (5′→3′)	Reverse primer (5′→3′)
PENK	ACTTGGTGCTGTTGTGCT	TTACCTTCCACTTCCATTAC
Ikaros	GACGCAACAATCCAACATAAC	ACGCCCATTCTCTTCATCA
GAPDH	ATGACTCTACCCACGGCAAG	CTGGAAGATGGTGATGGGTT

## Tissue Preparation and BrdU Immunohistochemistry

Following completion of the behavioral assessments, rats (N = 3) were deeply anesthetized with 10% chloral hydrate (1 mL/kg). Transcardial perfusion was performed through the left ventricle with 100 to 150 mL of normal saline followed by 200 to 250 mL of 4% paraformaldehyde in 0.1 M phosphate-buffered saline (PBS). The brains were removed, fixed in 4% paraformaldehyde, and embedded in paraffin for histological analysis.

Coronal brain sections (5 μm thickness) were prepared using a microtome, including sections containing the hippocampus. To identify transplanted MSC-derived cells, BrdU immunohistochemical staining was performed. Paraffin sections were deparaffinized and rehydrated, followed by antigen retrieval in citrate buffer at 65 °C for 2 hours. The sections were then washed with PBS and incubated in 2 mol/L HCl at 37 °C for 30 minutes to denature DNA.

After washing, the sections were permeabilized with 0.5% Triton X-100 for 20 minutes and incubated overnight at 4 °C with anti-BrdU primary antibody (Alexa Fluor 488 Goat Anti-Mouse IgG, BioLegend, USA). The sections were subsequently washed with PBS and incubated with FITC-conjugated secondary antibody (ab97022, 1:200, Abcam, UK). The stained sections were examined using a fluorescence microscope (Olympus Corporation, Japan). BrdU-positive cells were counted to determine the distribution and survival of transplanted cells.

## Immunohistochemistry for BDNF

The expression of brain-derived neurotrophic factor (BDNF) in the hippocampus was examined using immunohistochemistry. Brain sections were subjected to antigen retrieval by microwave heating in 0.01 M citrate buffer for 15 minutes. The sections were then incubated at 37 °C for 45 minutes in a blocking solution containing 10% normal goat serum and 3% Triton X-100 in PBS.

Subsequently, the sections were incubated overnight at 4 °C with a polyclonal anti-BDNF primary antibody (PA1-18371, Thermo Fisher Scientific, USA) diluted in PBS. After washing with PBS (three times for 5 minutes), the sections were incubated with FITC-conjugated anti-rabbit IgG secondary antibody (ab6717, 1:200, Abcam, USA) at room temperature for 2 hours.

For nuclear counterstaining, the sections were incubated with 4’,6-diamidino-2-phenylindole (DAPI, 1 µg/mL, Sigma-Aldrich) for 10 minutes at room temperature. Fluorescent images were acquired using an Olympus fluorescence microscope (Olympus Corporation, Tokyo, Japan). The percentage of BDNF-positive cells in the hippocampal CA1 subfield was quantified across five randomly selected microscopic fields per section by an investigator blinded to experimental conditions using ImageJ software (National Institutes of Health, Bethesda, MD, USA).

## Statistical Analyses

Statistical analyses were performed using GraphPad Prism (version 6.0). Data are expressed as mean ± standard error of the mean (SEM). The normality of the data distribution was evaluated using the Shapiro–Wilk test. For normally distributed data, one-way analysis of variance (ANOVA) followed by Tukey’s post-hoc test was employed. In instances where the normality assumption was not met or sample sizes were limited, non-parametric tests (Kruskal–Wallis followed by Dunn’s post-hoc test) were utilized. Differences were considered statistically significant at p < 0.05.

## Results

### Proenkephalin-Expressing Differentiated MSCs

Quantitative real-time PCR analysis was performed to evaluate the expression of proenkephalinergic MSCs markers in differentiated cells compared with undifferentiated MSCs. The results demonstrated a marked upregulation of PENK transcript levels in differentiated cells (144.85) relative to undifferentiated controls (4.75).

Similarly, the expression of the transcription factor Ikaros, which is involved in neuronal lineage specification, was significantly elevated in proenkephalin-expressing differentiated cells. The relative transcript level of Ikaros reached 31.95 in differentiated cells, whereas its expression remained minimal in undifferentiated MSCs (0.05). Statistical analysis confirmed that transcript levels of both PENK and Ikaros were significantly higher in differentiated cells compared with undifferentiated MSCs (*p* < 0.0001). Together, these findings confirm the successful differentiation of MSCs toward a proenkephalinergic phenotype, as evidenced by the robust upregulation of molecular markers associated with enkephalin signaling and neuronal lineage commitment (Figure 2).

**Figure 2.**
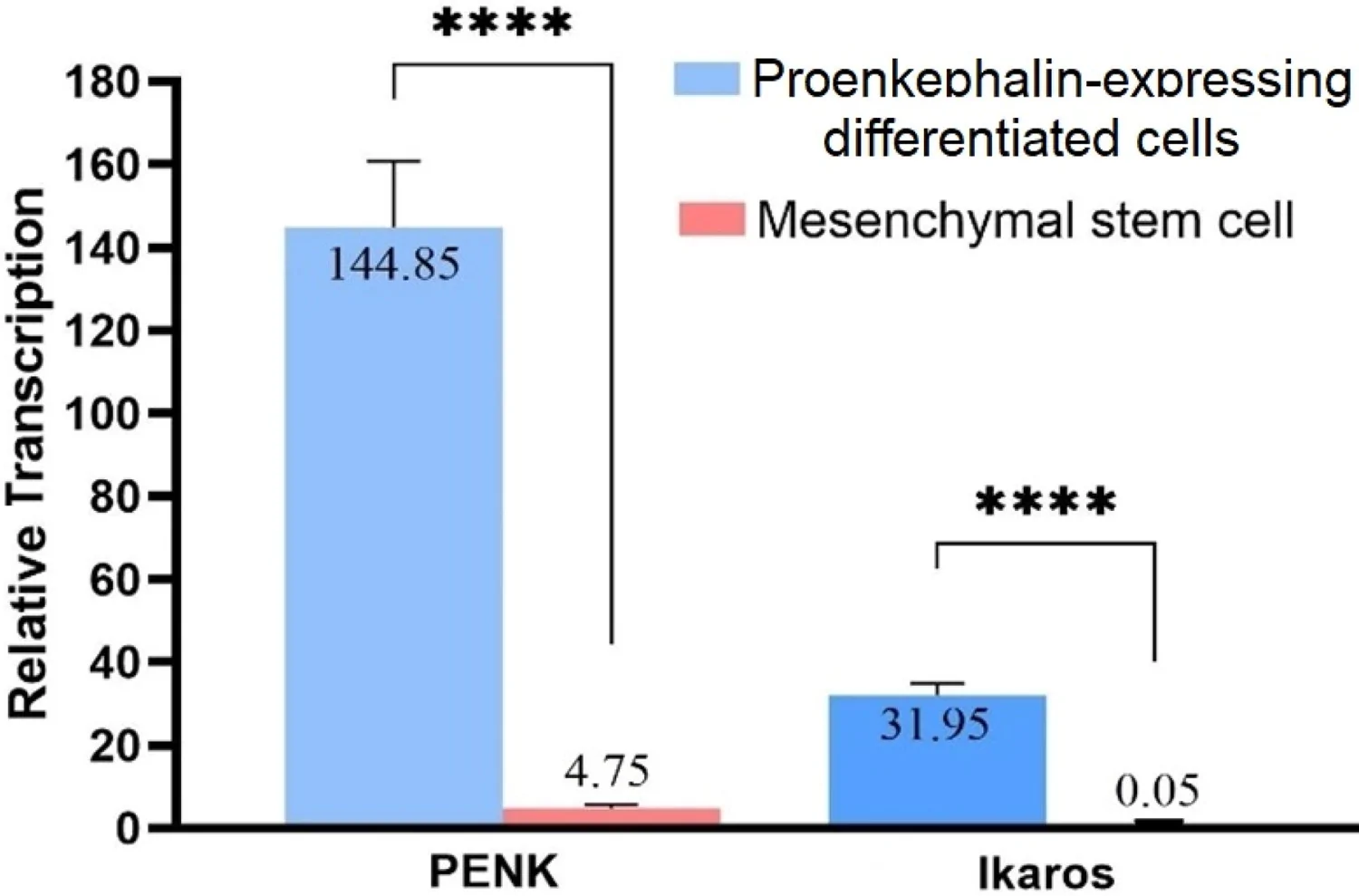
Relative gene expression of PENK and Ikaros in differentiated MSCs. Quantitative real-time PCR analysis was performed to evaluate the mRNA expression levels of proenkephalin (PENK) and the transcription factor Ikaros in differentiated cells compared with MSCs. Gene expression was analyzed using the ΔCt method and normalized to the housekeeping gene GAPDH. Data are presented as mean ± SEM of log2 fold change relative to the control group. Statistical analyses were conducted on ΔCt values. Differentiated cells showed a significant upregulation in PENK and Ikaros transcript levels compared with undifferentiated MSCs. ****p < 0.0001.

## Tracking of Transplanted Cells

To evaluate the migration and localization of transplanted cells, PENK-MSCs were pre-labeled with BrdU prior to administration. The labeled cells were delivered intranasally to animals in the treatment group. Fourteen days after transplantation, brain tissues were collected and examined using fluorescence microscopy to detect BrdU-positive cells in the hippocampus.

Microscopic observations revealed distinct BrdU-positive cells characterized by bright nuclear fluorescence with faint cytoplasmic labeling. Numerous BrdU-labeled cells were detected within the hippocampal region of ENK-treated rats, confirming successful intranasal migration and parenchymal localization of the delivered cells within the targeted brain area. The labeled cells appeared scattered throughout the hippocampal parenchyma, suggesting that intranasal administration enabled the cells to reach and distribute within the target region (Figure 3).

**Figure 3.**
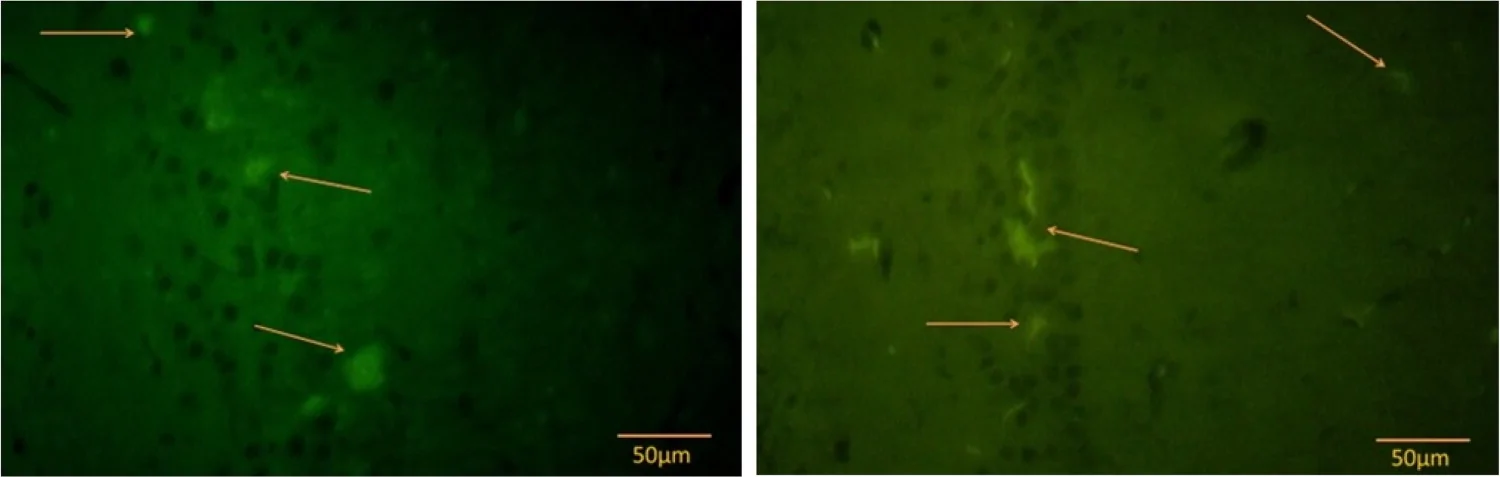
Detection of BrdU-labeled cells in the hippocampus following intranasal delivery. Proenkephalin-expressing differentiated cells were pre-labeled with BrdU and delivered intranasally to morphine-dependent rats. Fluorescence microscopy performed 14 days after administration revealed BrdU-positive cells within the hippocampal tissue. The labeled cells exhibited prominent nuclear fluorescence with faint cytoplasmic staining. Arrows indicate representative BrdU-positive cells distributed within the hippocampal region. Scale bar = 50 µm.

## Conditioned Place Preference

Following three days of morphine conditioning, rats developed a significant CPP, as evidenced by a marked increase in the time spent in the morphine-paired chamber during the first test session (CPP1). In CPP1, both the sham and PENK groups showed a significantly higher preference score compared with the control group (P < 0.01), confirming successful induction of morphine-associated place conditioning.

Following intranasal intervention, the second preference test (CPP2) revealed a significant reduction in time spent in the morphine-paired compartment in the PENK-treated group compared with the vehicle-treated sham group (*P* < 0.01, Figure 4).

**Figure 4.**
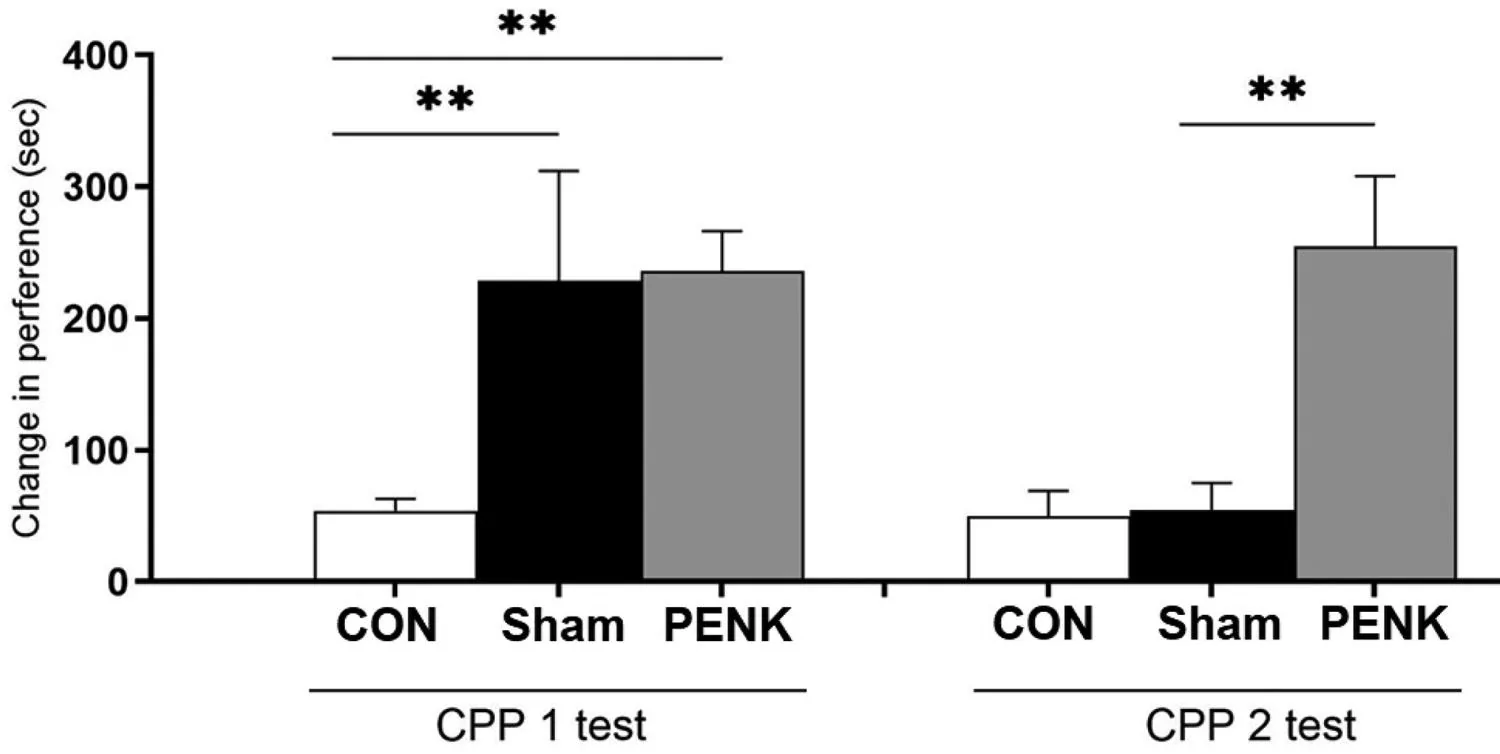
Effect of PENK treatment on morphine-induced CPP. Preference scores are presented as the change in time spent in the morphine-paired chamber (seconds) during the CPP tests. In the CPP1 test, sham and ENK groups showed significantly higher preference scores compared with the control group, indicating successful establishment of morphine-induced place preference. In the CPP2 test, ENK-treated rats exhibited a significant reduction in preference for the morphine-paired chamber compared with the sham group, suggesting attenuation of drug-seeking behavior. Data are presented as mean ± SEM (*n* = 8 per group). **P < 0.01. CON, control; CPP, conditioned place preference; Sham, morphine-treated sham group; PENK, treated with proenkephalin-expressing differentiated cells.

## Novel Object Recognition Test

No significant difference was observed among groups in total exploration time (Figure 5A), suggesting that alterations in discrimination index were not due to differences in exploration activity. Morphine-treated (sham) rats exhibited a significant reduction in discrimination index (DI) compared with the control group (*P* < 0.0001, Figure 5B), indicating impaired recognition memory following morphine exposure. In contrast, the PENK-treated group demonstrated a significant improvement in DI compared with the morphine-treated vehicle group (*P* < 0.01, Figure 5B).

**Figure 5.**
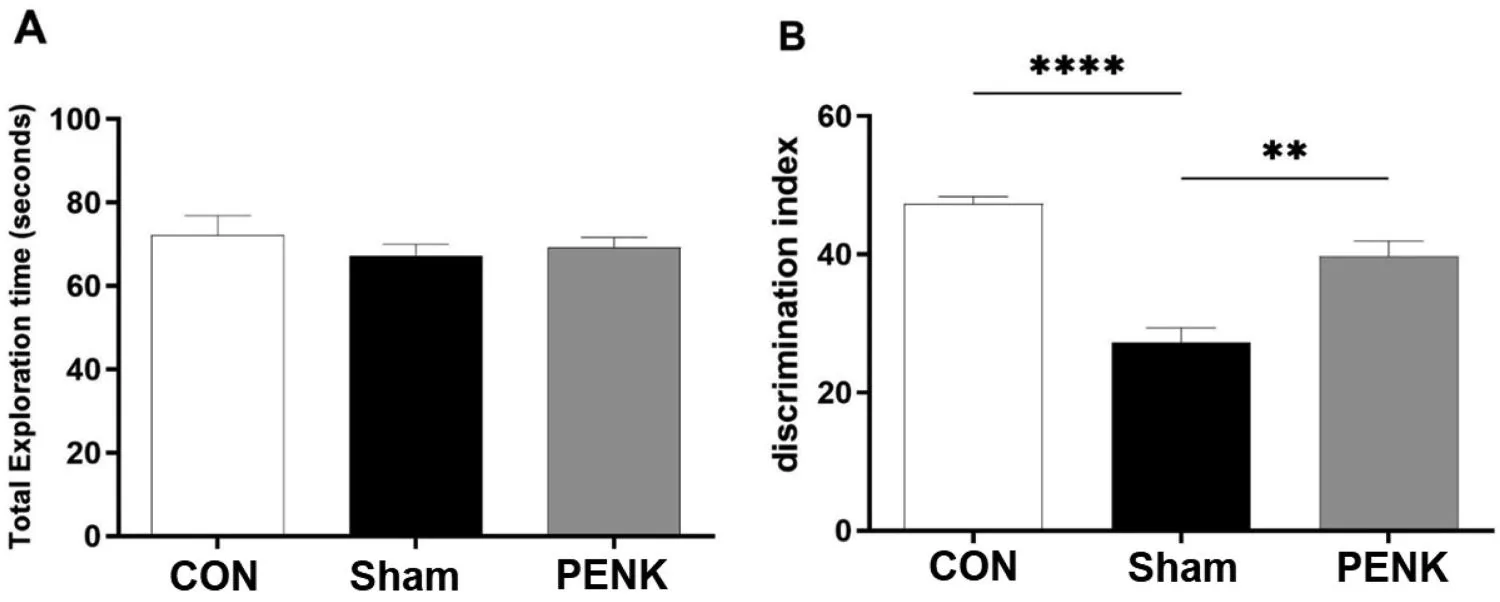
Effect of PENK treatment on novel object recognition performance after morphine administration. (A) Total exploration time: the total time (seconds) spent exploring both the familiar and novel objects during the test phase. (B) Discrimination index (DI): calculated as (time exploring novel object − time exploring familiar object)/(total exploration time for both objects). A decrease in DI indicates recognition memory impairment. Sham group showed a significant reduction in DI compared with controls, whereas ENK-treated rats displayed a significant improvement in DI, consistent with partial restoration of recognition memory. Data are expressed as mean ± SEM (n = 8 per group). ****P < 0.0001, **P < 0.01. CON, control; Sham, morphine-treated sham group; PENK, treated with proenkephalin-expressing differentiated cells.

## Elevated Plus Maze

Behavioral assessment using the EPM revealed significant anxiety-like behavior in morphine-treated rats. Compared with the control group, morphine-dependent (sham) rats spent significantly less time in the open arms (P < 0.01; Figure 6A) and exhibited fewer entries into the open arms (P < 0.01; Figure 6B), indicating increased anxiety-like behavior following morphine exposure. Treatment with PENK-MSCs significantly increased both the time spent in the open arms and the number of open-arm entries compared with morphine-treated vehicle group (P < 0.01; Figure 6A, B).

**Figure 6.**
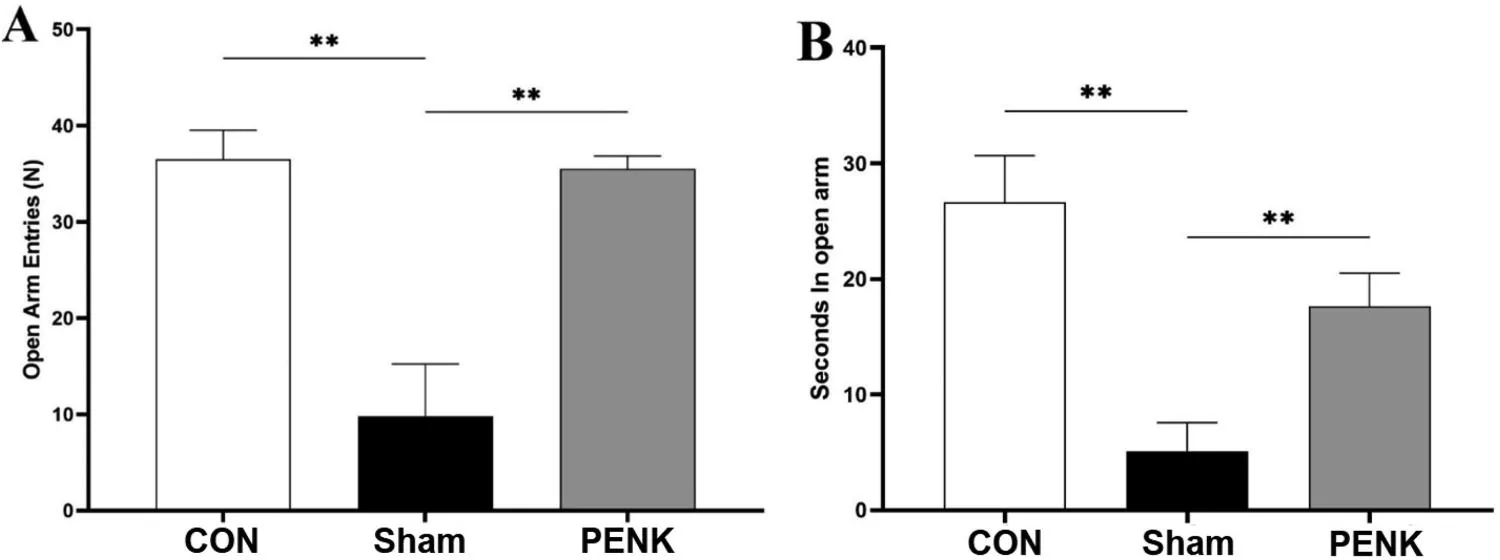
Effects of PENK treatment on anxiety-like behavior in the elevated plus maze following morphine exposure. (A) number of entries into the open arms and (B) Time spent in the open arms were measured as indicators of anxiety-related behavior. Sham) group showed significantly reduced open-arm exploration compared with the control group, reflecting increased anxiety-like behavior. ENK treatment significantly increased both the duration and frequency of open-arm exploration compared with the sham group, suggesting an anxiolytic effect. Data are expressed as mean ± SEM (*n* = 8 per group). **P < 0.01. CON, control; Sham, morphine-treated sham group; PENK, treated with proenkephalin-expressing differentiated cells.

## BDNF Expression in Hippocampus

Analysis of BDNF immunoreactivity in the hippocampus revealed that morphine-dependent (sham) rats exhibited significantly reduced BDNF levels compared with the control group (*P* < 0.001; Figure 7). Treatment with PENK-MSCs markedly increased hippocampal BDNF expression compared with the sham group (P < 0.01; Figure 7).

**Figure 7.**
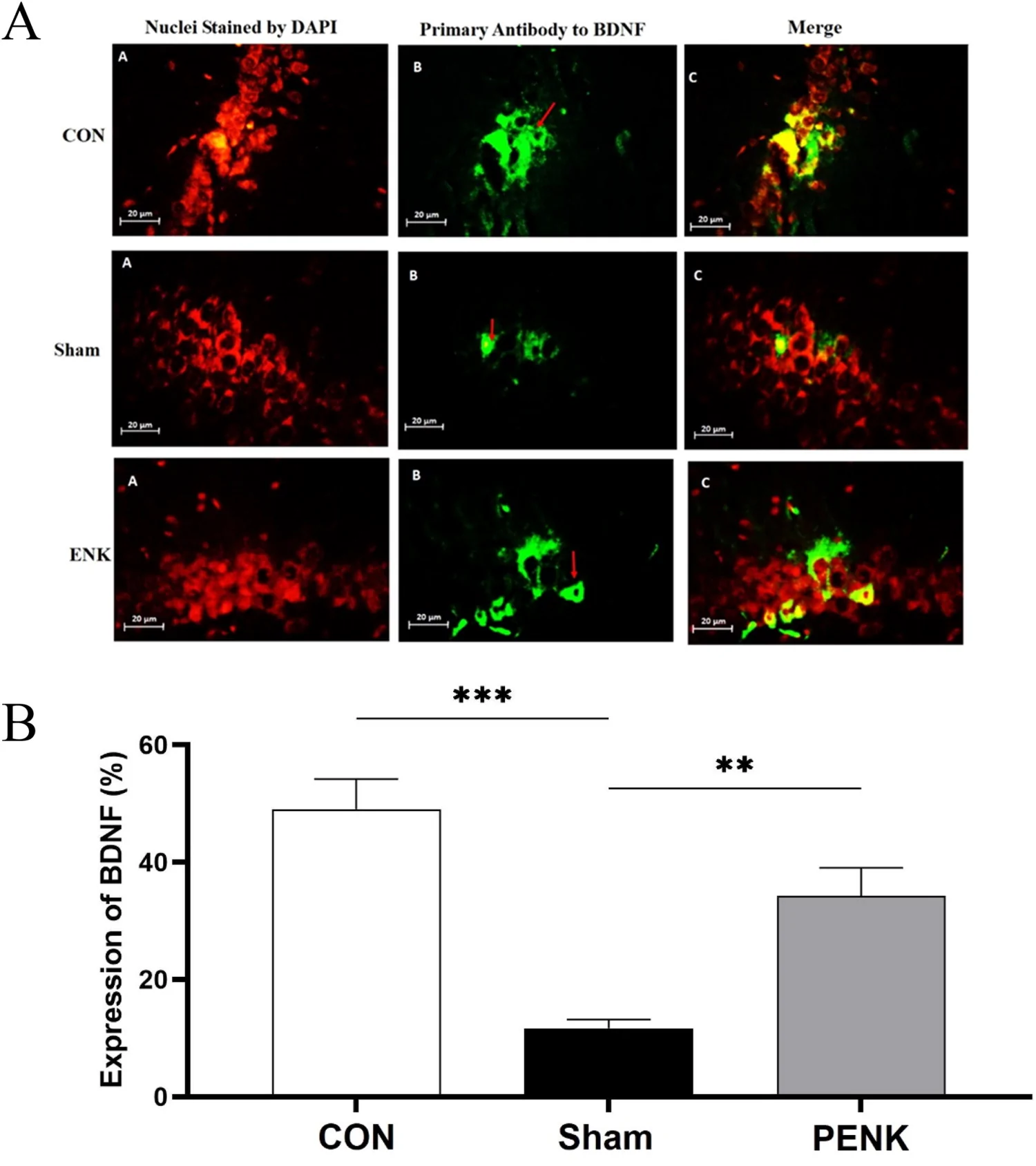
Immunohistochemical analysis of BDNF expression in the hippocampus after morphine exposure and ENK treatment. (A) Representative images showing BDNF immunoreactivity and nuclear counterstaining with DAPI in the hippocampus of CON, sham, and ENK-treated groups. Merged images are shown in the right panel of each group; scale bar = 20 µm. (B) Quantitative analysis of the percentage of BDNF-positive cells in the hippocampal CA1 region. Morphine dependence significantly reduced BDNF levels, whereas ENK administration significantly restored BDNF expression. Data are expressed as mean ± SEM (*n* = 3 per group). **P < 0.01, ***P < 0.001. CON, control; Sham, morphine-treated sham group; BDNF, brain-derived neurotrophic factor; PENK, treated with proenkephalin-expressing differentiated cells.

## Discussion

The present study aimed to investigate whether intranasal delivery of PENK-MSCs could attenuate behavioral and neurobiological alterations associated with morphine dependence in a rat model. Overall, the findings demonstrated that administration of PENK-MSCs produced significant improvements in addiction related behavioral and neurobiological outcomes. Behavioral assessments indicated that morphine exposure induced marked impairments in reward related behavior, recognition memory, and anxiety like responses, whereas treatment with PENK-MSCs significantly ameliorated these alterations. Specifically, animals receiving cell administration showed reduced morphine associated preference in the CPP paradigm, improved performance in the NOR test, and reduced anxiety like behavior in the EPM. In addition to these behavioral effects, histological analysis revealed significant neurobiological change, including restoration of hippocampal BDNF expression following PENK-MSCs administration.

The CPP findings of the present study showed that morphine administration produced a significant increase in drug associated preference, indicating the successful establishment of morphine related reward learning. Importantly, transplantation of PENK-MSCs significantly attenuated morphine induced CPP, suggesting a reduction in drug seeking behavior and reward related memory. These findings are consistent with previous studies demonstrating that administration of morphine can influence reward processing and drug associated learning (Gheibi et al., 2025). Experimental evidence indicates that enkephalins regulate dopaminergic neurotransmission within the mesolimbic pathway, particularly in projections from the ventral tegmental area to the nucleus accumbens, which play a central role in mediating drug reward (L. G. Rysztak & E. M. Jutkiewicz, 2022). Enhanced enkephalin signaling has been reported to modulate dopamine release and alter the motivational properties of addictive substances (Mongi-Bragato et al., 2018). In line with the present results, several studies have shown that manipulation of opioid peptides or their receptors can reduce addictive behavior (Wee & Koob, 2010). For instance, experimental studies have reported that modulation of delta opioid receptors, which are activated by enkephalins, can influence reward related learning and reduce the reinforcing effects of addictive substances (Nieto et al., 2005; Pellissier et al., 2018). In interpreting the functional recovery observed in this study, a critical biochemical distinction must be made between elevated proenkephalin (PENK) transcript abundance and the definitive release of biologically active mature peptides. While immature or proliferative lineages can transiently express prepropeptide transcripts, our 12-day directed neural induction protocol yielded a robust concomitant upregulation of neural commitment factors alongside PENK elevation, indicative of lineage-specific differentiation. In committed neural phenotypes, proenkephalin undergoes sequential post-translational processing by prohormone convertases (PC1/3 and PC2) and carboxypeptidase E to generate bioactive Met- and Leu-enkephalin.

In addition to reward related behavior, the present study also demonstrated that morphine exposure impaired cognitive performance and increased anxiety like behavior, as evidenced by reduced discrimination performance in the NOR test and decreased open arm exploration in the EPM. These findings are consistent with previous reports indicating that chronic opioid exposure disrupts hippocampal dependent memory processes and promotes anxiety related behaviors (Gheibi et al., 2025). Opioid induced neuroadaptations within hippocampal and limbic circuits, including alterations in glutamatergic transmission and neurotrophic signaling, have been implicated in these behavioral impairments (Hosseinzadeh Sahafi et al., 2023). Notably, administration of PENK-MSCs significantly improved recognition memory and reduced anxiety like behavior in morphine exposed animals. These results are in agreement with previous studies suggesting that modulation of endogenous opioid signaling and restoration of neural network homeostasis may contribute to improvements in cognitive and emotional functions following chronic drug exposure (Olusakin & Lobo, 2023; Pecina & Zubieta, 2018; Toubia & Khalife, 2019).

The observed increase in hippocampal BDNF expression following administration of PENK-MSCs is in line with a substantial body of literature implicating BDNF as a critical mediator of synaptic plasticity, learning, and emotional regulation in the context of SUDs (Estrada-Medina et al., 2026). Previous studies have reported that chronic opioid exposure is associated with region specific dysregulation of BDNF signaling, particularly within the hippocampus and other limbic structures, which has been linked to impairments in memory processing and heightened vulnerability to relapse (Krukru et al., 2026; Nazari et al., 2025). In agreement with the present findings, experimental interventions that restore neurotrophic support, including cell-based therapies, environmental enrichment, or pharmacological modulation, have been shown to normalize BDNF levels and improve behavioral outcomes related to cognition and affect (Nazari et al., 2025; Rafaiee et al., 2023). Conversely, some studies have reported that increased BDNF signaling in mesolimbic regions such as the nucleus accumbens may facilitate drug seeking and relapse, (Collo et al., 2014) highlighting the region dependent and context specific effects of BDNF in addiction related behaviors. This regional specificity may provide a plausible explanation for the beneficial behavioral effects observed and underscores the importance of considering brain region, timing, and mode of intervention when interpreting the role of BDNF in addiction related neuroplasticity.

It is noteworthy to evaluate whether the observed behavioral and neurotrophic changes could reflect non-specific neuroinflammatory responses or cytokine-induced sickness behavior secondary to allogeneic cell administration. Pro-inflammatory cytokine surges typically produce profound behavioral suppression, characterized by reduced overall locomotor exploration and marked anhedonia (Varela et al., 2025). In our assessments, total exploration time in the NOR paradigm remained entirely preserved, while open-arm exploration in the EPM was significantly elevated, a pattern directly arguing against sickness behavior or generalized motor depression. Furthermore, although acute neuroinflammatory insults can transiently upregulate neurotrophins, sustained subacute neuroinflammation consistently suppresses hippocampal BDNF transcription and impairs synaptic plasticity. Therefore, the concurrent restoration of hippocampal BDNF expression and memory discrimination on post-transplantation day 14 reflects constructive neuroplastic recovery rather than a persistent inflammatory injury cascade.

Immunohistochemical analysis and tracking of BrdU labeled cells provided direct evidence for the survival, targeted migration, and parenchymal localization of transplanted cells within the target tissue. The presence of BrdU positive cells in different regions of the hippocampus indicated that the intranasally administered PENK-MSCs were able to successfully bypass anatomical and physiological barriers and reach multiple brain regions. This observation is consistent with previous reports demonstrating that the intranasal route can facilitate the direct delivery of cells and bioactive molecules to the central nervous system, (Lu et al., 2022; Rafaiee et al., 2023) highlighting the translational relevance of this non-invasive administration pathway.

Mechanistically, the robust upregulation of proenkephalin achieved prior to transplantation suggests that the observed behavioral and neurotrophic recovery is driven by local neuropeptidergic rebalancing rather than diffuse, non-specific stem cell actions. Chronic opiate exposure induces profound dysregulation of endogenous opioid signaling, characterized by mu-opioid receptor desensitization and homeostatic decoupling within reward circuitry. In this context, localized enkephalinergic supplementation via intranasally delivered differentiated cells may function as a physiological buffer. By restoring basal delta- and mu-opioid receptor activation, graft-derived enkephalins could dampen the compensatory hyper-reactivity of the mesolimbic dopamine system during withdrawal and extinction, while simultaneously promoting mood stabilization and anxiolysis. Furthermore, the persistence of functional recovery and elevated hippocampal BDNF expression at post-transplantation day 14 is consistent with a paracrine-mediated hit-and-run mode of action rather than permanent cellular engraftment. Under this framework, intranasally administered cells exert rapid trophic and peptide-mediated signaling during their transient residence, triggering enduring downstream neuroplastic remodeling that outlasts the physical clearance of the graft.

Several methodological considerations of the present study should be acknowledged. First, because this investigation was designed as an initial proof-of-concept study, analytical assessments focused primarily on in vitro transcriptional validation and in vivo downstream behavioral and neurotrophic endpoints. Consequently, the relative contribution of lineage-specific opioid signaling versus the broader, non-specific paracrine secretome was not experimentally segregated in the current design. Recognizing these structural boundaries establishes the proof-of-principle nature of our findings while providing a precise framework for subsequent mechanistic validation.

Building on these foundational findings, future investigations should pursue several mechanistic and translational avenues. First, incorporating genetically edited donor cells, such as proenkephalin-knockout lines generated via CRISPR/Cas9, alongside central opioid receptor antagonists will definitively isolate peptide-mediated effects from basal stem cell actions. Second, quantitative profiling of mature Met- and Leu-enkephalin concentrations alongside region-specific mapping of opioid receptor subtypes across mesolimbic and corticolimbic circuits will provide fine-grained neurochemical resolution. Third, comprehensive multiplex screening of central and systemic inflammatory cytokines will clarify how immunomodulatory and neuroplastic cascades intersect during recovery. Finally, longitudinal studies incorporating non-invasive in vivo cell tracking, extended abstinence and relapse paradigms, and dose-optimization protocols are needed to determine whether therapeutic effects persist after opioid withdrawal, characterize long-term cell fate and safety, and establish whether booster dosing is required.

## Conclusion

In summary, the present study demonstrates that intranasal delivery of PENK-MSCs attenuated addiction-related behavioral alterations and improved cognitive performance in a morphine-dependent rat model. PENK-MSCs treatment mitigated anxiety-like behavior, enhanced recognition memory, and upregulated hippocampal BDNF expression. These findings suggest that treatment with PENK-MSCs may be a feasible experimental approach for modulating neurobiological pathways involved in SUDs. Nevertheless, further investigations are required to clarify the long-term efficacy, underlying molecular mechanisms, and translational relevance of this approach before clinical considerations.

## Data Availability

The data that support the findings of this study are available from the corresponding author upon reasonable request.

## References

[CIT0001] Ali, A. H., Ahmed, H. S., Jawad, A. S., & Mustafa, M. A. (2021). Endorphin: Function and mechanism of action. *Science Archives*, *2*(1), 9–13. doi.

[CIT0002] Badshah, I., Anwar, M., Murtaza, B., & Khan, M. I. (2024). Molecular mechanisms of morphine tolerance and dependence; novel insights and future perspectives. *Molecular and Cellular Biochemistry*, *479*(6), 1457–1485. doi. 10.1007/s11010-023-04810-3 37470850

[CIT0003] Camarini, R. (2019). Mesenchymal stem cells as new perspective for the treatment of alcohol use disorder. *Gene Therapy*, *27*(10-11), 471–473. 10.1038/s41434-020-0133-0 32107474

[CIT0004] Collo, G., Cavalleri, L., & Spano, P. (2014). Structural plasticity in mesencephalic dopaminergic neurons produced by drugs of abuse: Critical role of BDNF and dopamine. *Frontiers in Pharmacology*, *5*, 259. doi. 10.3389/fphar.2014.00259 PMC4243500 25505416

[CIT0005] Estrada-Medina, R., Briones-Llamoctanta, B. E., & Turpo-Chaparro, J. E. (2026). Neuroplasticity and recovery of the brain affected by substance use disorder: Multilevel mechanisms and new therapeutic strategies (2020–2025). *Frontiers in Molecular Neuroscience*, *19*, 1760387. doi. 10.3389/fnmol.2026.1760387 PMC12979374 41836311

[CIT0006] Gheibi, M., Nazari, S., Mohseni, F., Salehi Sangani, P., Felehkari, F., Niknamfar, S., Ghazvini, H., Seyedhosseini Tamijani, S. M., Kalalian-Moghaddam, H., Jomehpour, H., & Rafaiee, R. (2025). Coenzyme Q10 ameliorates anxiety and depression‑like behavior associated with chronic opioid use and increases GDNF expression in the hippocampus of morphine‑dependent rats. *Acta Neurobiologiae Experimentalis*, *85*(2), 94–104. 10.55782/ane-2025-2581 40742011

[CIT0007] Ghelardini, C., Mannelli, L. D. C., & Bianchi, E. (2015). The pharmacological basis of opioids. *Clinical Cases in Mineral and Bone Metabolism: The Official Journal of the Italian Society of Osteoporosis, Mineral Metabolism, and Skeletal Diseases*, *12*(3), 219–221. doi. 10.11138/ccmbm/2015.12.3.219 PMC4708964 26811699

[CIT0008] Hafizi, M., Bakhshandeh, B., Soleimani, M., & Atashi, A. (2012). Exploring the enkephalinergic differentiation potential in adult stem cells for cell therapy and drug screening implications. *In Vitro Cellular & Developmental Biology. Animal*, *48*(9), 562–569. doi. 10.1007/s11626-012-9546-4 23054438

[CIT0009] Hosseinzadeh Sahafi, O., Sardari, M., Alijanpour, S., & Rezayof, A. (2023). Shared mechanisms of GABAergic and opioidergic transmission regulate corticolimbic reward systems and cognitive aspects of motivational behaviors. *Brain Sciences*, *13*(5), 815. doi. 10.3390/brainsci13050815 PMC10216078 37239287

[CIT0010] Koob, G. F., & Volkow, N. D. (2016). Neurobiology of addiction: A neurocircuitry analysis. *The Lancet. Psychiatry*, *3*(8), 760–773. 10.1016/s2215-0366(16)00104-8 PMC6135092 27475769

[CIT0011] Koob, G. F., Kandel, D. B., Baler, R. D., & Volkow, N. D. (2023). *Neurobiology of addiction*. Tasman’s Psychiatry (pp. 1–51). Springer.

[CIT0012] Krukru, E. E., Chinko, B. C., & Dapper, V. D. (2026). Opioid-induced dysregulation of BDNF signalling: A study of acute, sub-chronic, and chronic exposure in a rodent model. *Biomedical Sciences*, *12*(1), 10–16. doi. 10.11648/j.bs.20261201.12

[CIT0013] Listos, J., Łupina, M., Talarek, S., Mazur, A., Orzelska-Górka, J., & Kotlińska, J. (2019). The mechanisms involved in morphine addiction: An overview. *International Journal of Molecular Sciences*, *20*(17), 4302. doi. 10.3390/ijms20174302 PMC6747116 31484312

[CIT0014] Lu, S., Li, K., Yang, Y., Wang, Q., Yu, Y., Wang, Z., & Luan, Z. (2022). Optimization of an intranasal route for the delivery of human neural stem cells to treat a neonatal hypoxic-ischemic brain injury rat model. *Neuropsychiatric Disease and Treatment*, *18*, 413–426. doi. 10.2147/NDT.S350586 PMC9047963 35495583

[CIT0015] Melemis, S. M. (2023). *Relapse and relapse prevention. Alcohol use: Assessment, withdrawal management, treatment and therapy: Ethical practice* (pp. 349–361). Springer.

[CIT0016] Mohseni, F., Rahimi, K., Niroumand Sarvandani, M., Jamali, Z., Seyedhosseini Tamijani, S. M., & Rafaiee, R. (2022). Lapse and relapse rates in narcotics anonymous versus methadone maintenance treatment: A 12-month prospective study. *Iranian Journal of Psychiatry*, *17*(1), 1–13. 10.18502/ijps.v17i1.8044 PMC8994838 35480125

[CIT0017] Mongi-Bragato, B., Avalos, M. P., Guzmán, A. S., Bollati, F. A., & Cancela, L. M. (2018). Enkephalin as a pivotal player in neuroadaptations related to psychostimulant addiction. *Frontiers in Psychiatry*, *9*, 222. doi. 10.3389/fpsyt.2018.00222 PMC5985699 29892236

[CIT0018] Nazari, S., Gheibi, M., Salehi Sangani, P., Felehkari, F., Gohardehi, F., Niknamfar, S., Jomehpour, H., Ghazvini, H., Seyedhosseini Tamijani, S. M., Vahabzadeh-Kebria, M., Mohseni, F., & Rafaiee, R. (2025). Coenzyme Q10 suppressed cue-related reinstatement through cognition improvement and hippocampal increases BDNF expression in morphine-dependent male rats. *Basic and Clinical Neuroscience*, *16*(2), 379–392. 10.32598/bcn.2024.1425.7 PMC13318235 42382507

[CIT0019] Nieto, M. M., Guen, S., Kieffer, B., Roques, B., & Noble, F. (2005). Physiological control of emotion-related behaviors by endogenous enkephalins involves essentially the delta opioid receptors. *Neuroscience*, *135*(2), 305–313. doi. 10.1016/j.neuroscience.2005.06.025 16112476

[CIT0020] Olusakin, J., & Lobo, M. K. (2023). An endogenous opioid alters neuronal plasticity to constrain cognitive flexibility. *Molecular Psychiatry*, *28*(8), 3146–3148. doi. 10.1038/s41380-023-02204-x PMC10859914 37532796

[CIT0021] Pecina, M., & Zubieta, J.-K. (2018). Expectancy modulation of opioid neurotransmission. *International Review of Neurobiology*, *138*, 17–37. doi. 10.1016/bs.irn.2018.02.003 PMC6314670 29681324

[CIT0022] Pellissier, L. P., Pujol, C., Becker, J., & Le Merrer, J. (2018). Delta opioid receptors: Learning and motivation. *Handbook of Experimental Pharmacology*, *247*,.227–260. doi. 10.1007/164_2016_89 28035528

[CIT0023] Rafaiee, R., Ahmadiankia, N., Mousavi, S. A., Jafari, B., & Kalalian Moghaddam, H. (2023). Bone marrow mesenchymal stem cells improve cognitive impairments induced by methamphetamine in rats and reduce relapse. *BioImpacts: BI*, *13*(2), 97–108. 10.34172/bi.2022.23329 PMC10182440 37193077

[CIT0024] Ranaiy, M. S., Ghazvini, H., Tamijani, S. M. S., Yazdian-Robati, R., Ahmadiankia, N., & Rafaiee, R. (2024). Implanting sinoatrial node-like pacemaker cells into the substantia nigra of the brain as a novel therapeutic approach for Parkinson’s disease. *Medical Hypotheses*, *189*, 111419. 10.1016/j.mehy.2024.111419

[CIT0025] Rysztak, L. G., & Jutkiewicz, E. M. (2022). The role of enkephalinergic systems in substance use disorders. *Frontiers in Systems Neuroscience*, *16*, 932546. 10.3389/fnsys.2022.932546 PMC9391026 35993087

[CIT0026] Shirasaka, T., Hashimoto, E., Ukai, W., Yoshinaga, T., Ishii, T., Tateno, M., & Saito, T. (2012). Stem cell therapy: Social recognition recovery in a FASD model. *Translational Psychiatry*, *2*(11), e188. 10.1038/tp.2012.111 PMC3565770 23149452

[CIT0027] Toubia, T., & Khalife, T. (2019). The endogenous opioid system: Role and dysfunction caused by opioid therapy. *Clinical Obstetrics and Gynecology*, *62*(1), 3–10. doi. 10.1097/GRF.0000000000000409 30398979

[CIT0028] Varela, R. B., Macpherson, H., Walker, A. J., Houghton, T., Yates, C., Yates, N. J., Daygon, V. D., & Tye, S. J. (2025). Inflammation and metabolic dysfunction underly anhedonia-like behavior in antidepressant resistant male rats. *Brain, Behavior, and Immunity*, *127*, 170–182. doi. 10.1016/j.bbi.2025.03.001 40064431

[CIT0029] Volkow, N. D., & Blanco, C. (2023). Substance use disorders: A comprehensive update of classification, epidemiology, neurobiology, clinical aspects, treatment and prevention. *World Psychiatry: Official Journal of the World Psychiatric Association (WPA)*, *22*(2), 203–229. doi. 10.1002/wps.21073 PMC10168177 37159360

[CIT0030] Wee, S., & Koob, G. F. (2010). The role of the dynorphin–κ opioid system in the reinforcing effects of drugs of abuse. *Psychopharmacology*, *210*(2), 121–135. doi. 10.1007/s00213-010-1825-8 PMC2879894 20352414

[CIT0031] Xiong, M., Tao, Y., Gao, Q., Feng, B., Yan, W., Zhou, Y., Kotsonis, T. A., Yuan, T., You, Z., Wu, Z., Xi, J., Haberman, A., Graham, J., Block, J., Zhou, W., Chen, Y., & Zhang, S.-C. (2021). Human stem cell-derived neurons repair circuits and restore neural function. *Cell Stem Cell*, *28*(1), 112–126.e6.e116. doi. 10.1016/j.stem.2020.08.014 PMC7796915 32966778

[CIT0032] Yang, H.-Y., Wu, X.-M., Liu, Y., & He, D. (2015). Transplantation of bone marrow mesenchymal stem cells promotes learning and memory functional recovery and reduces hippocampal damage in rats with alcohol-associated dementia. *Transplantation*, *99*(3), 492–499. doi. 10.1097/TP.0000000000000535 25695788

